# Impact of COVID-19 on excess mortality, life expectancy, and years of life lost in the United States

**DOI:** 10.1371/journal.pone.0256835

**Published:** 2021-09-01

**Authors:** Eunice Y. S. Chan, Davy Cheng, Janet Martin

**Affiliations:** 1 Centre for Medical Evidence, Decision Integrity & Clinical Impact (MEDICI), Department of Anesthesia & Perioperative Medicine, Schulich School of Medicine & Dentistry, Western University, London, Ontario, Canada; 2 Department of Epidemiology & Biostatistics, Schulich School of Medicine & Dentistry, Western University, London, Ontario, Canada; Istituto di Ricerche Farmacologiche Mario Negri, ITALY

## Abstract

This paper quantifies the net impact (direct and indirect effects) of the pandemic on the United States population in 2020 using three metrics: excess deaths, life expectancy, and total years of life lost. The findings indicate there were 375,235 excess deaths, with 83% attributable to direct, and 17% attributable to indirect effects of COVID-19. The decrease in life expectancy was 1.67 years, translating to a reversion of 14 years in historical life expectancy gains. Total years of life lost in 2020 was 7,362,555 across the USA (73% directly attributable, 27% indirectly attributable to COVID-19), with considerable heterogeneity at the individual state level.

## Introduction

With more than 375,000 COVID-19 deaths in the year 2020, the United States have been heavily affected by the pandemic. Due to the high number of COVID-19 deaths and limited health resources especially early in the pandemic, many healthcare resources were diverted to prioritize treating COVID-19 patients. This has garnered many concerns that treatment for other common diseases has been neglected and thus, has attributed to the increase in time-sensitive non-COVID-19 deaths. Therefore, we planned to quantify the impact that the coronavirus has had on the population of the United States, not just the overall impact, but also the impact attributable to the direct and indirect effects of the pandemic, separately. We used three global metrics in health to quantify the impact that COVID-19 has had in the United States: excess mortality, life expectancy (LE), and years of life lost (YLL). Excess mortality refers to the number of deaths that exceeds what would have been observed under “normal conditions”. Life expectancy is used for assessing population health in which it tells us the average age of death in a population. YLL estimates the years of life lost due to premature deaths. We also define the impact on population health due to COVID-19 deaths alone as direct effects, and the impact attributable to other causes as indirect effects of COVID-19.

There have been several studies that have examined the impact of the COVID-19 pandemic in the United States, which also quantified the impact using global health metrics. The impact of the pandemic on excess mortality on the entire population of the United States has been studied extensively, including reports from the CDC, Financial Times, Our World in Data, and published articles by Woolf et al., and Islam et al. [1–5]. On the other hand, there have been fewer studies on the impact of pandemic on life expectancy, including published articles by Andrasfay and Goldman, and Woolf, Masters, and Aron [6, 7]. At present, there have been no studies that examined the years of life lost due to the COVID-19 pandemic to our knowledge. There have also been no studies that quantified and compared the direct and indirect impact of the pandemic in the United States for the entirety of the year 2020 which address any of these three population metrics. This study aims to fill these gaps in the literature.

Using the provisional COVID-19 death count [8] published by the Centers for Disease Control and Prevention (CDC), we approximated: 1) number of excess deaths; 2) number of years in life expectancy decreased and the number of years reverted in historical life expectancy gains, and; 3) YLL attributed to the direct and indirect effects of COVID-19 for the total, male, and female population in the United States (at both the country and state level) in the year 2020. In this manuscript, we will focus on the impact of COVID-19 in the United States at the country level. For results at the individual state-level, see the app at: https://medicimagic.shinyapps.io/Impact_of_COVID-19_in_USA/.

## Methods

### Data sources

Data for this analysis was obtained from two CDC data sets: “Provisional COVID-19 Death Counts by Sex, Age, and State,” and “Underlying Causes of Death (1999–2019)” [8, 9]. “Underlying Causes of Death (1999–2019)” was used to extrapolate the potential number of deaths in 2020 that would have occurred if COVID-19 had not impacted the population. We grouped the data by ten-year age groups (< 1 year, 1–4 years, 5–14 years, 15–24 years, 25–34 years, 35–44 years, 45–54 years, 55–64 years, 65–74 years, 75–84 years, and 85+ years), state, and by year, and calculated any suppressed data cells for both data sets (see S1 Appendix for more details).

### Calculating excess mortality

To estimate the number of excess deaths attributed to the overall effects of COVID-19 in the year 2020, rather than comparing the reported number of deaths to the mean number of deaths reported for the corresponding week over the previous five years (which numerous researchers [2, 3, 10] have done to estimate excess mortalities) or using Farrington surveillance algorithms to determine the number of excess deaths (which is what the CDC uses) [1], we compared the total number of reported deaths to the results extrapolated from all available data on the number of deaths published by CDC Wonder (past 21 years) for each age group. This extrapolated result represents our estimate on the number of deaths that would have occurred under “normal” conditions if the pandemic had not occurred.

In order to calculate excess mortality from all causes, we first determined a baseline number of deaths that would have occurred in 2020 under “normal” circumstances by linearly regressing the annual number of deaths from 1999–2019 using a 95% prediction interval. We then subtracted this baseline from the reported total deaths for all age groups according to sex and state in order to calculate excess all-cause mortality attributed to COVID-19.

In this study, we assumed that all reported COVID-19 deaths were excess deaths attributable to the direct effects of the pandemic. Even though we know that this count may not necessarily be accurate as there were challenges in identifying deaths caused by COVID-19, especially early in the pandemic, we believe that this is a conservative estimate on the direct effects of the pandemic. To calculate the excess deaths attributed to the indirect effects of the disease, we subtracted the number of COVID-19 deaths from the excess deaths attributed to the overall effects of the disease.

### Calculating decrease in life expectancy

#### Abridged life tables

To estimate the decrease in life expectancy, we first estimated three life expectancies: the reference, actual, and non-COVID-19 life expectancy using an abridged life table. In this study, we used an abridged life table rather than a complete life table due to the fact that the CDC published their provisional COVID-19 data by 10-year age groups rather than by single year. An abridged life table is a data-driven tool that summarizes the probability of a person dying before entering the next age cohort. These probabilities allow us to calculate the life expectancy at birth using a series of calculations represented in columns in the life table. Table 1 presents the notation, definition, and formula for each column. To determine the life expectancy, we require two inputs: the number of deaths and the mid-year population. These two values make up the annual death rate and will change according to the life expectancy that we are calculating.

**Table 1:** Notation, definition, and formula of each column of an abridged life table to calculate life expectancy.

Notation	Definition	Formula
(*x*, *x*+*n*)	Age interval or period of life between two exact age stated in years	
_*n*_ *m*_*x*_	Annual death rate	$\frac{\mathrm{Number}\;\mathrm{of}\;\mathrm{Deaths}}{\mathrm{Mid}‐\mathrm{year}\;\mathrm{Population}}$
_*n*_ *q*_*x*_	Proportion of persons alive at the beginning of the age interval who die during the age interval	1−exp(−*n*⋅_*n*_*m*_*x*_)
*I*_*x*_	Of the starting number of newborns in the life table, the number of living at the beginning of the age interval	*I*_*x*+*n*_ = *I*_*x*_−_*n*_*d*_*x*_
_*n*_ *d*_*x*_	The number of persons in the cohort who died in the age interval (*x*, *x*+*n*)	*I*_*x*_⋅_*n*_*q*_*x*_
_*n*_ *L*_*x*_	Number of years of life lived by the cohort within the indicated age interval (*x*, *x*+*n*)	$\frac{{}_{n}d_{x}}{{}_{n}m_{x}}$
*T*_*x*_	Total person-years of life contributed by the cohort after attaining age *x*	$\sum_{x}^{\mathrm{End}\;\mathrm{of}\;\mathrm{table}}{}_{n}L_{x}$
$e_{x}^{0}$	Average number of years of life remaining for a person alive at the beginning of age interval *x*	$\frac{T_{x}}{I_{x}}$

#### Establishing reference, actual, and non-COVID-19 life expectancy in 2020

To estimate the decrease in life expectancy attributed to the direct and indirect effects of COVID-19 in the United States at the country level, by state, and for total, male and female populations, we first established a reference, actual, and non-COVID-19 life expectancy. The reference life expectancy is our estimate on what the life expectancy would have been in the United States in 2020 without the pandemic. The actual life expectancy is calculated based on the total number of deaths (with and without COVID-19) that occurred in the year 2020. Lastly, the non-COVID-19 life expectancy is calculated based on the number of non-COVID-19 deaths in the United States, reported by the CDC. For all three life expectancy calculations, we provide the fitted, lower and upper bounds of our results based on the 95% prediction intervals from our annual death count and mid-year population estimates. For the fitted life expectancy, we used the fitted value from both the annual number of deaths and mid-year population estimates. For the lower bound, we used the upper bound of the annual number of deaths and the lower bound of the mid-year population estimates. This combination of number of deaths and mid-year population gives us the greatest possible death rate, and therefore, the lower bound of the life expectancy. Conversely, for the upper bound, we used the lower bound of the annual number of deaths and the upper bound of the mid-year population.

*Reference life expectancy*. The reference life expectancy is to establish a baseline of what the life expectancy would have been if the pandemic had not occurred. Since there is no way of knowing what the number of deaths would have been for the United States in 2020 without the pandemic, we estimated the number of annual deaths that would have occurred by using the same method (linear regression) as we previously did when estimating the baseline number of deaths to determine the excess mortality attributable to the COVID-19 pandemic, along with a 95% prediction interval for each age and sex strata. On the other hand, since at the time of writing the CDC has not published the mid-year population for the year 2020, we also estimated this value by regressing the mid-year population data set from 1990 to 2019 using a cubic polynomial for every grouping of age, sex and state with a 95% prediction interval for each group. These estimates for the annual number of deaths and the mid-year population were used to calculate the reference life expectancy for the entire country as well as each state and for total, male and female populations.

*Actual life expectancy*. The actual life expectancy is our estimate on what the life expectancy was in the United States based on the total number of deaths reported by the CDC. For the numerator of the death rate (annual number of deaths), we used the total deaths reported by the CDC, and for the denominator of the death rate (mid-year population), we used a revised mid-year population estimate to approximate life expectancy during the pandemic in the United States and for each state for the total, male, and female populations. We modified the mid-year population to account for excess mortality attributable to COVID-19 by taking the estimated mid-year population (from the previous section) and subtracting it by half of the excess mortality estimated from the previous section.

*Non-COVID-19 life expectancy*. The non-COVID-19 life expectancy estimates the life expectancy based on the non-COVID-19 deaths that occurred in the United States in 2020. This calculation allows us to determine the proportion of the decrease in life expectancy that is attributed to the direct effects of the COVID-19 pandemic. For the non-COVID-19 life expectancy calculations, we used the number of non-COVID-19 deaths for our numerator and the extrapolated mid-year population for our denominator. Similar to the previous two life expectancy calculations, we estimated the life expectancy for the United States, each state, and for the total, male and female population.

A summary of the values used for the numerator (annual number of deaths) and the denominator (mid-year population) of the death rate can be found in Table 2.

**Table 2:** Summary of the values used for life expectancy calculations.

LE Calculation	Annual Deaths	Mid-Year Population
Reference	Baseline # of deaths (extrapolated using cubic regression)	Estimated mid-year population (extrapolated using cubic regression)
Actual	Total number of deaths in 2020 reported by the CDC	Estimated mid-year population minus half of excess deaths
Non-COVID-19	Number of non-COVID-19 deaths in 2020 reported by the CDC	Estimated mid-year population

#### Estimating the decrease in life expectancy during the pandemic

To calculate the decrease in life expectancy due to the pandemic, we took the difference between the life expectancies calculated in the previous section in various combinations. To calculate the decrease in life expectancy due to the overall effects of the pandemic, we subtracted the reference life expectancy by the actual life expectancy; for the decrease in life expectancy due to the direct effects of the pandemic, we subtracted the non-COVID-19 life expectancy by the actual life expectancy; and for the decrease in life expectancy due to the indirect effects of the pandemic, we subtracted the reference life expectancy by the non-COVID-19 life expectancy. The formulas to calculate the decrease in life expectancy attributed to the overall, direct and indirect effects are summarized below:

DecreaseinLifeExpectancy(OverallEffects)=ReferenceLE−ActualLE

DecreaseinLifeExpectancy(DirectEffects)=Non‐COVID‐19LE−ActualLE

DecreaseinLifeExpectancy(IndirectEffects)=ReferenceLE−Non‐COVID‐19LE

Additionally, we determined the number of years reverted in historical life expectancy gains. Since the life expectancy values given by the CDC [11] are calculated using a complete life table rather than an abridged life table, we recalculated the life expectancies for the total, male, and female population of the United States (the entire country and for each state) from 1999 to 2019 using an abridged life table to create historical life expectancy values that are better comparable to the life expectancies that we calculated for the year 2020. By using the same method to calculate the historical life expectancy, this allows us to attain a better approximation on the number of years lost in historical life expectancy gains. We then compared the life expectancies that we calculated for the year 2020 to historical life expectancy values for each region and sex and determined the most recent year in which the life expectancy was similar to the present one.

### Calculating years of life lost

In order to calculate the years of life lost attributed to the direct and indirect effects of the coronavirus, we used the following formula:

$$\mathrm{YLL}=\sum_{i}^{N}{\left(\mathrm{Reference}\;\mathrm{Life}\;\mathrm{Expectancy}\right)}_{i}\times {\left(\mathrm{Excess}\;\mathrm{Mortality}\right)}_{i}$$

where *N* is the number of age cohorts, and *i* corresponds to the *i*-th age cohort. In words, we took the sum of the product between the number of excess deaths by the reference life expectancy for each respective age cohort. To calculate the overall, direct, and indirect effect of the COVID-19 pandemic on YLL, we used the corresponding excess mortality that we calculated in the previous section. We also used the reference life expectancy established earlier in this paper in these calculations.

For a more detailed description of the methodologies, see S1 Appendix.

## Results

In the following, we summarize the results for United States at the country level for the total, male, and female population. For the results for each state, see S1 Appendix.

### Excess mortality

**Fig 1:**
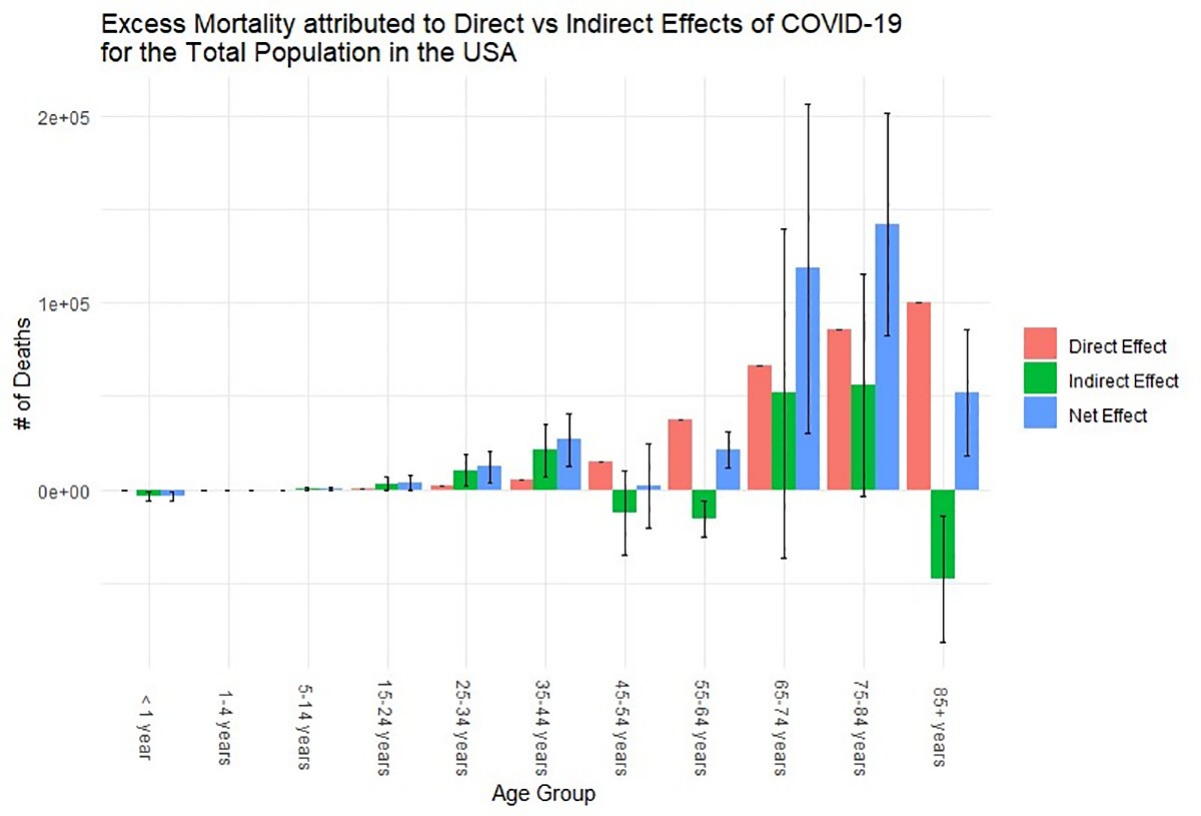
Excess mortality attributable to direct vs indirect effects of COVID-19 in 2020. Error bars indicate the lower and upper bounds of the plausible range of excess mortality.

Our findings suggest that there was a total of 375,235 excess deaths (95% PI: 132,380, 618,088), with 83% attributed to direct and 17% attributed to indirect effects of the COVID-19 pandemic in the year 2020. The male population of the United States had an excess of 220,457 deaths (59% of the fitted number of excess deaths), whereas the female population had an excess of 154,669 deaths (41% of the total number of excess deaths). We also observed that the direct and indirect effects of the pandemic affected the male and female population differently: for the male population, 77% of the excess deaths were attributed to the direct effects of the pandemic; on the other hand, for the female population, 93% of excess deaths are attributed to the direct effects of the pandemic. Fig 1 compares the number of excess deaths attributed to the direct and indirect effects of COVID-19 for the total population of the United States stratified by age group. We observed that those between the ages of 5 and 44 were heavily affected due to the indirect effects of COVID-19 in comparison to the direct effects. On the other hand, those who are above the age of 44 were more affected by COVID-19 itself rather than other causes of death.

### Life expectancy

**Fig 2:**
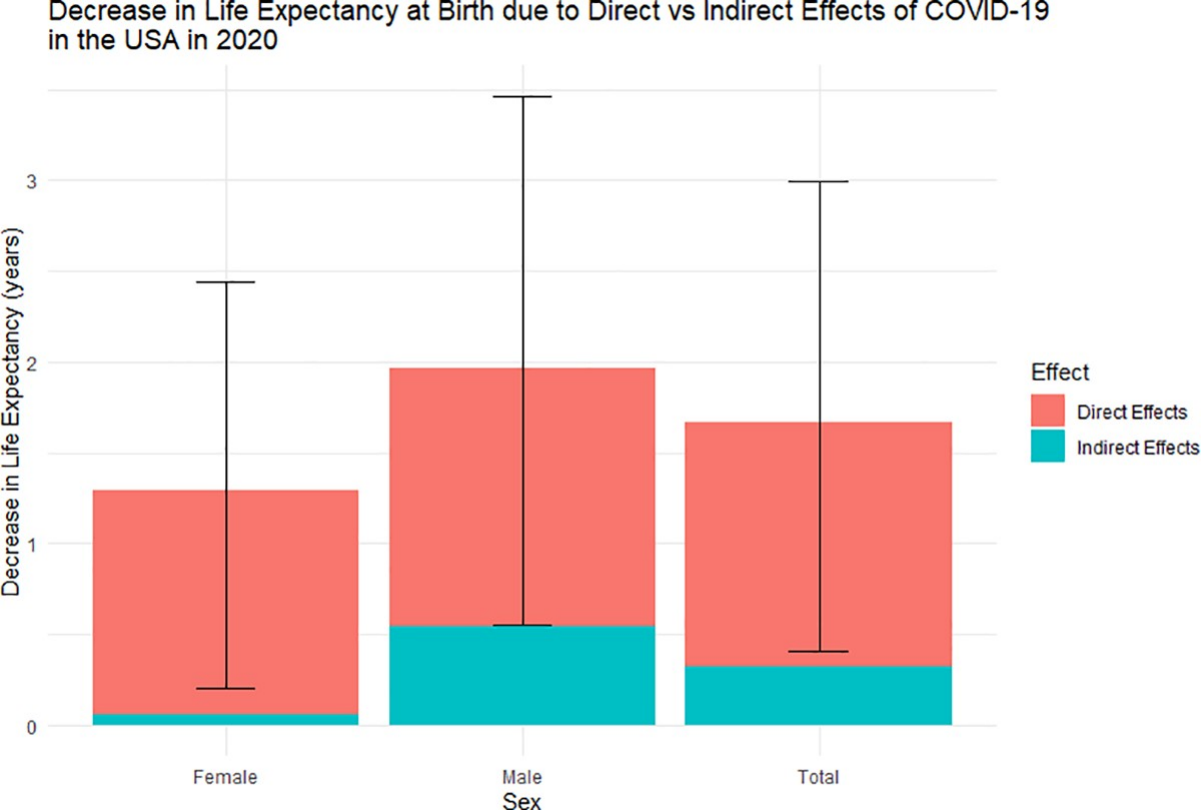
Number of years lost in life expectancy due to the direct and indirect effects of COVID-19 on the female, male, and total population of the United States in 2020. Error bars are the uncertainty of the overall effects of COVID-19 for each respective sex.

The increase in the number of deaths in the United States due to the COVID-19 pandemic resulted in the life expectancy at birth to decrease. Fig 2 shows the decrease in life expectancy attributed to the direct and indirect effects of COVID-19 for the male, female, and total population of the United States. For the total population, the life expectancy decreased by 1.67 (0.41–3.0) years, in which 1.35 years (81% of overall effects) were attributed to the direct effects of the disease. This decrease in life expectancy due to the overall impact of the pandemic translates to the reversion of approximately 14 (12–17) years in historical life expectancy gains. Comparing the impact COVID-19 has had on life expectancy for the male and female population, we observed that both sexes were heavily affected by the pandemic: the life expectancy of males decreased 1.96 years (which is approximately 15 years lost in historical life expectancy gains), and the life expectancy of females decreased 1.29 years (which is approximately 12 years lost in historical life expectancy gains). Although both sexes were heavily affected by the direct effects of COVID-19, males were more affected by the indirect effects of COVID-19 compared to their female counterparts (28% of the life expectancy decrease for the male population was attributed to the indirect effects of the disease, compared to 4% for the female population).

### Years of life lost

**Fig 3:**
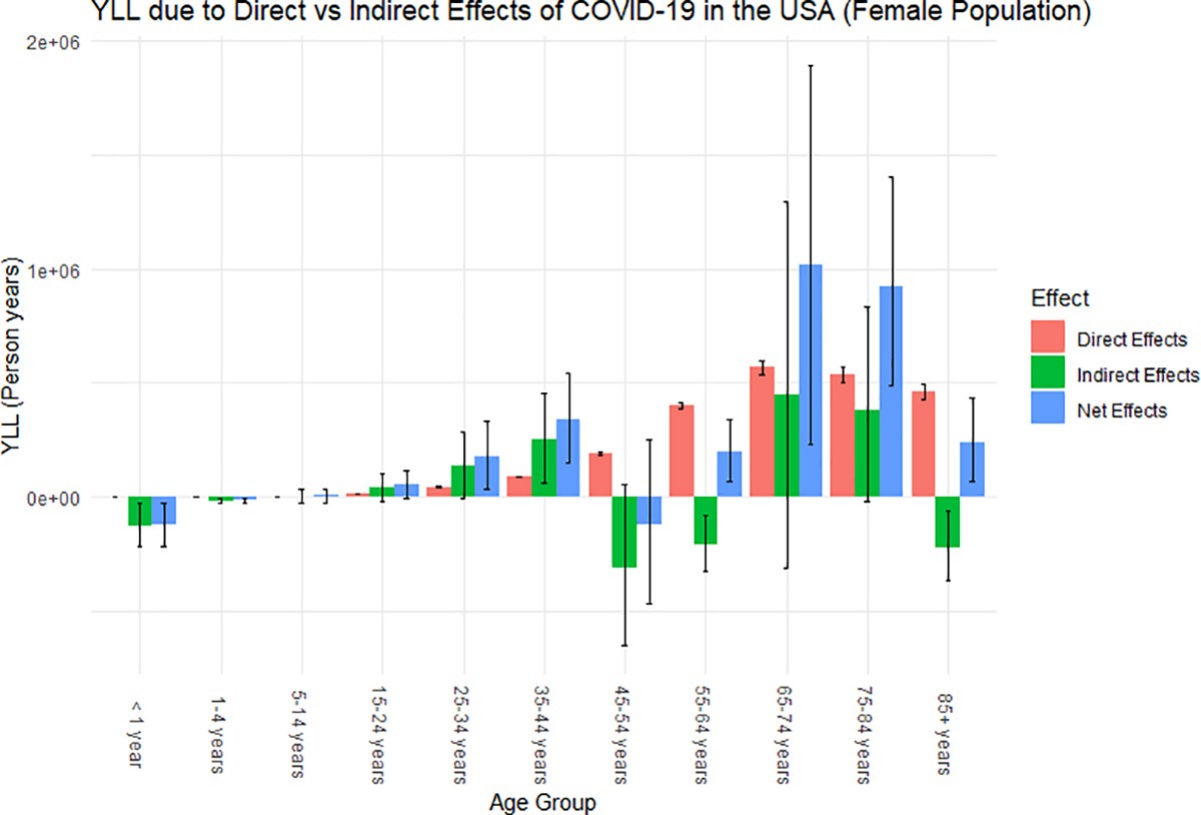
Years of life lost attributed to the overall effects of COVID-19 for the female population of the United States in 2020. Error bars are the uncertainty of how each effect impacted the YLL for each age group.

**Fig 4:**
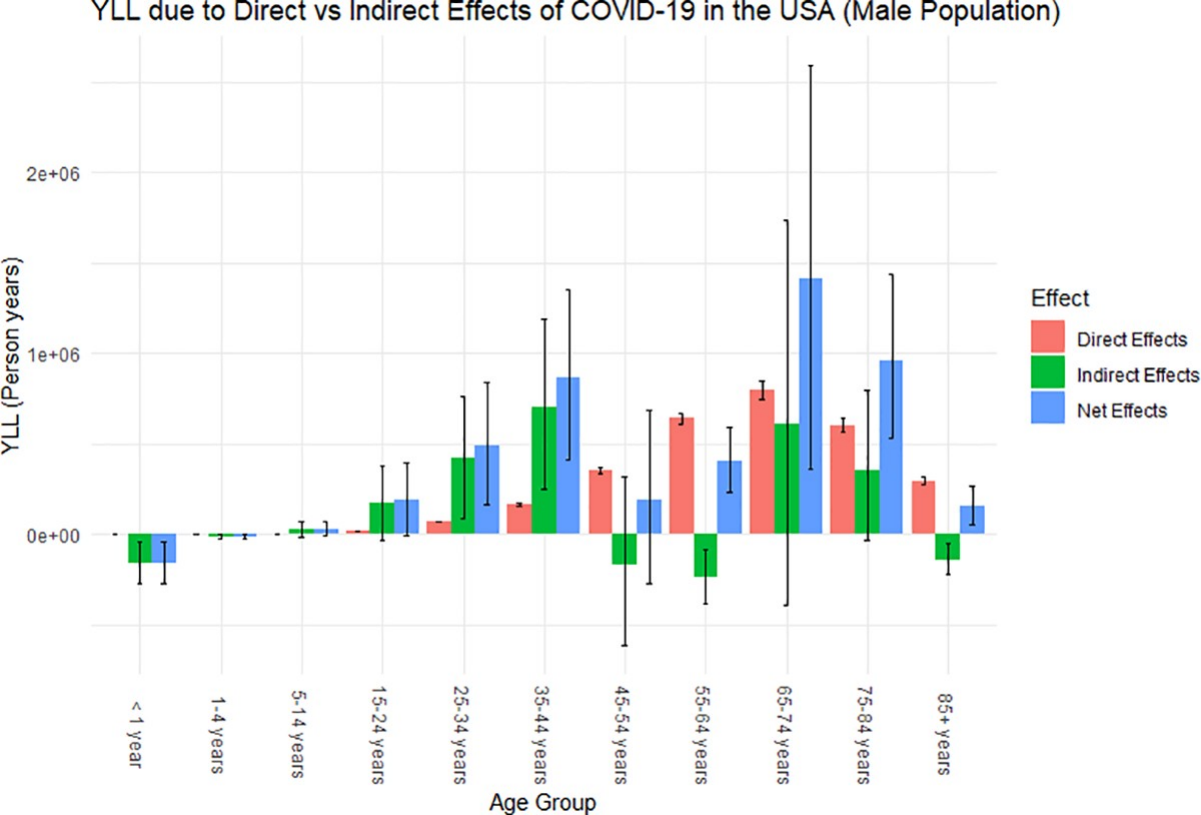
Years of life lost attributed to the overall effects of COVID-19 for the male population of the United States in 2020. Error bars are the uncertainty of how each effect impacted the YLL for each age group.

Overall, the YLL attributed to COVID-19 in the United States is approximately 7,362,555 person-years (95% PI: 1,596,202, 13,669,696), with 73% of those YLL directly attributed and 27% indirectly attributed (collateral effects leading to excess deaths during the pandemic) to COVID-19 (Table 3). The male population make up 61% of the total YLL (65% of which are due to the direct effects of the pandemic), whereas the female population make up 39% of the total YLL (86% of which are attributable to the direct effects of the pandemic). The distribution of the years of life lost attributed to the direct and indirect effects of COVID-19 across each age cohort for the female and male population can be observed in Figs 3 and 4, respectively.

**Table 3:** Summary of the direct, indirect, and overall effects of the COVID-19 pandemic in the United States in 2020.

	Excess Mortality	Life Expectancy Loss	YLL
**Direct Effects**	313171	1.35 (1.35, 1.35)	5,340,469 (5,068,888, 5,627,895)
**Indirect Effects**	62064 (-180791, 304917)	0.32 (-0.94, 1.64)	2,022,086 (-3472687, 8041802)
**Overall Effect**	375235 (132380, 618088)	1.67 (0.41, 2.99)	7,362,555 (1,596,202, 13,669,696)

Values in parentheses represent the 95% prediction interval.

From Figs 3 and 4, we observed similar trends between the male and female population in the distribution of YLL across the age cohorts. Similar to what was observed for excess mortality, for both the male and female population, those who are between the age of 15 and 44 were affected more by the indirect effects of the pandemic, whereas those who are above the age of 44 were affected more by the direct effects of the pandemic. Since we are examining YLL, meaning we are putting more weight on the younger population in our calculations, this amplifies the indirect impact of the COVID-19 pandemic on the overall population since the younger population is greatly affected by the collateral effects of the pandemic compared to the direct effects. In particular, we observe that males between the ages of 15 and 44 heavily contributed to the YLL attributable to the indirect effects of disease from Fig 4. This potentially attributed to the large decrease in life expectancy for the total and male population.

## Discussion

In this manuscript, we quantified both the direct and indirect effects of COVID-19 in the United States through three metrics: excess mortality, life expectancy, and years of life lost. This is the first manuscript, to our knowledge, to compare the total number of deaths directly and indirectly attributed to COVID-19 with the number of deaths for each age group extrapolated from the past 21 years. It is also the first to quantify the decrease in life expectancy and reversion of historical life expectancy gains for the male, female, and total population of the United States; and, to estimate the years of life lost attributed to direct and indirect effects of COVID-19.

Our findings suggest that there was a total of 375,235 excess deaths (83% attributable to direct and 17% attributable to indirect effects of COVID-19); a decrease in life expectancy by 1.67 (0.41–3.0) years (1.35 years directly attributable COVID-19), translating to the reversion of approximately 14 (12–17) years in historical life expectancy gains; and approximately 7,362,555 YLL (72.5% YLL directly attributed and 27.5% indirectly attributed). We also observed that the population between the ages of 15 and 44 are more affected by the indirect effects of the COVID-19 pandemic, whereas the population above the age of 44 are more affected by the direct effects of the pandemic.

**Fig 5:**
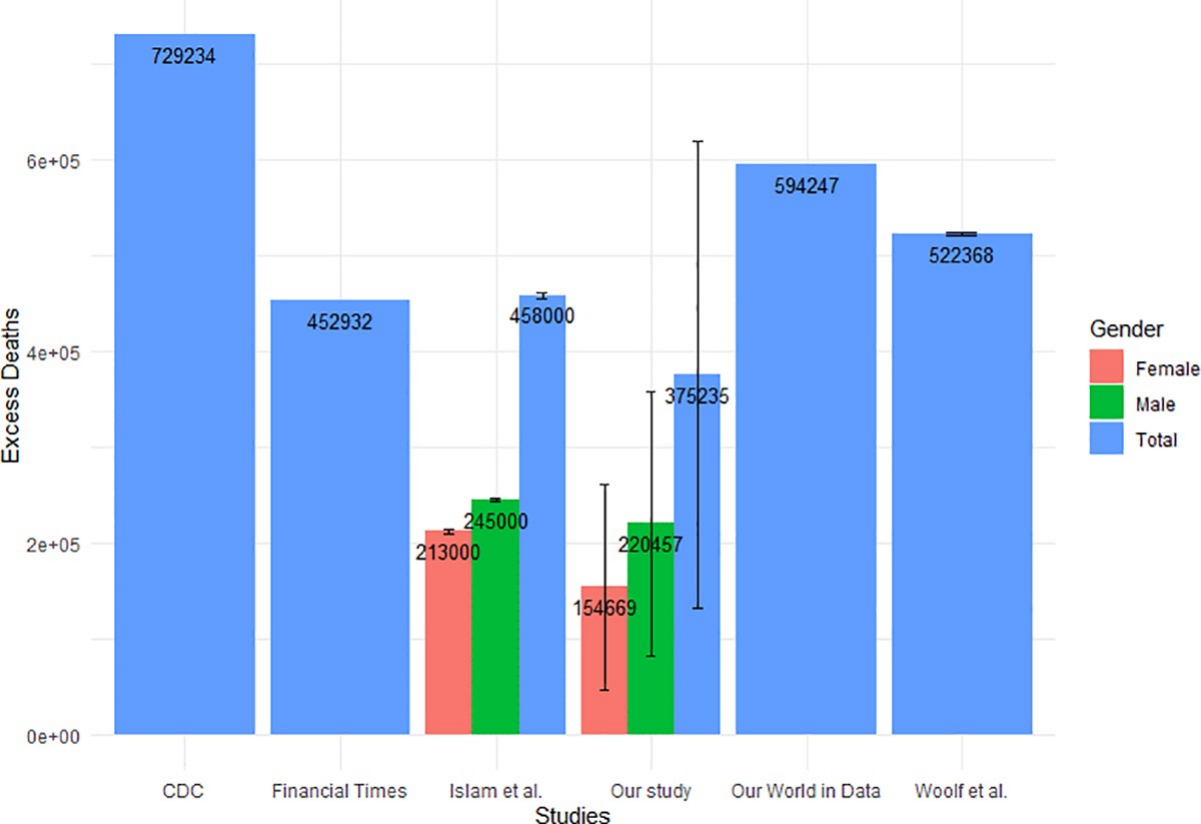
Comparison of excess mortality results attributable to COVID-19 pandemic in the United States in 2020 across different studies. Error bars are the uncertainty of how each effect impacted the excess mortality for each population.

Despite using different methodologies to calculate excess mortality, the results from our study show similar results to other related studies. Fig 5 compares the excess mortality attributed to the COVID-19 pandemic in the United States in the year 2020 across different studies. The CDC [1] used the Farrington surveillance algorithms in which “a range of values for the number of excess deaths was calculated as the difference between the observed count and one of the two thresholds by week and jurisdiction”. The Financial Times [2] and Our World in Data [3] both used the raw number of deaths observed over a period of a week during the COVID-19 pandemic and subtracted the average deaths over the same week over the previous five years. Lastly, Woolf et al. [4] and Islam et al. [5] both estimated the weekly number of excess deaths by using a Poisson regression model; however, Woolf et al. used mortality data from 2014–2019, while Islam et al. used mortality data from 2016–2019. We observed that the CDC gave the greatest estimate on the number of excess deaths; this is most likely due to the fact that they set all negative values (when calculating the weekly excess deaths) to zero, and therefore, the negative values were excluded from the number of excess deaths in the United States for the year 2020. On the other hand, our study gave the smallest estimate out of all the studies. This is possibly due to the use of linear regression, which potentially led to overestimating the baseline number of deaths that would occur in the year 2020 if the pandemic did not occur.

**Fig 6:**
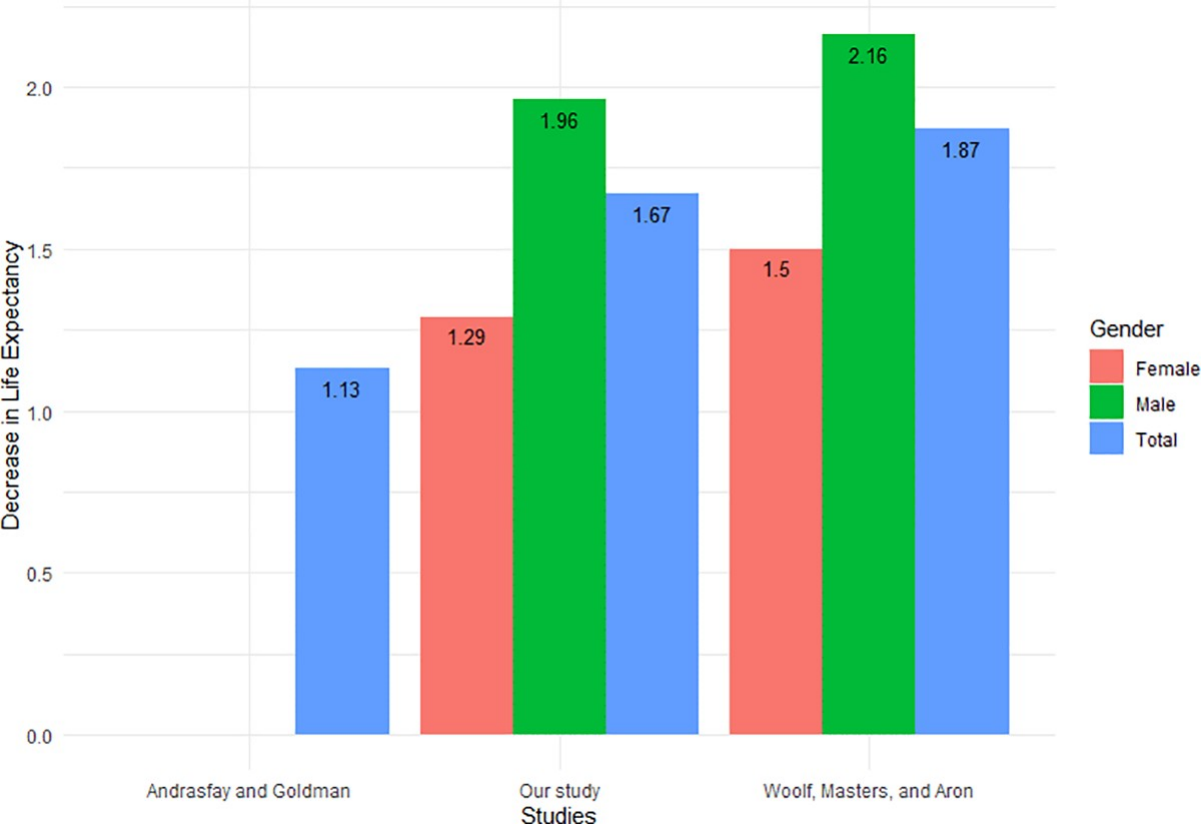
Comparison of life expectancy decrease attributable to COVID-19 pandemic in the United States in 2020 across different studies.

Fig 6 compares the decrease in life expectancy between our study and two other studies: Andrasfay and Goldman, and Woolf, Masters, and Aron [6, 7]. All three studies used a life table to calculate the current life expectancy, but the methods differ in the reference that they compared the current life expectancy to. Andrasfay and Goldman calculated the expected number of deaths from causes other than COVID-19 by using the age-specific mortality rates from 2017 and applying it to the 2019 population count by age group in the United States, which was then used to create a reference life expectancy value. They compared their reference life expectancy to three Institute for Health Metrics and Evaluation (IHME) projections based on different levels of COVID-19 mortality. The result presented in Fig 6 gives the medium scenario. As the pandemic comes in waves and the true number of deaths is not known, this may potentially skew the projections to underestimate the number of deaths due to COVID-19 in 2020, which may lead to an underestimation of the decrease in life expectancy. On the other hand, Woolf, Masters, and Aron compared their results with the life expectancy of the United States in 2018. Since Woolf, Masters, and Aron compared with 2020 life expectancy estimates, they could potentially be overestimating the life expectancy decrease, as they did not take the gain in life expectancy that could have occurred between 2018 and 2020. Although the authors mentioned that the life expectancy increase between the years 2018 and 2019 were only 0.1 years, we observe that our estimates on the decrease in life expectancy only differs from their study by 0.2 years. This suggests that historical life expectancy gains can potentially contribute to the overall decrease in life expectancy due to the COVID-19 pandemic. Our estimate falls between the estimates presented by the other two studies, which suggests that our results are reasonable.

The values for the excess mortality, number of years in life expectancy decreased, and the YLL presented in this manuscript should be interpreted in light of the following limitations. Since official mid-year populations for the United States (and for each respective state) for the year 2020 have not been released, we estimated these values for each age cohort using regression, along with a range of plausible values using 95% prediction intervals for our estimates. This resulted in a larger uncertainty interval for our excess mortality, life expectancy decrease, and YLL. Also, since we defined all COVID-19 deaths as excess deaths attributable to the direct effects of COVID-19, this may not necessarily give us an accurate depiction of how significant COVID-19 was relative to other deaths since there were challenges in identifying deaths due to the virus, especially early on the in the pandemic. Despite this, it does not change the results of the overall impact of the pandemic in our study but would only affect the proportion that is attributed to the direct effects of the pandemic. Additionally, due to our assumption that all COVID-19 deaths are excess deaths due to the direct effects of the pandemic, this occasionally results in negative numbers of excess mortality (and YLL) attributed to indirect effects of the disease. A few possible reasons why we see negative indirect effects include: the possibility that some COVID-19 deaths were not correctly diagnosed and reported; a number of people who died from COVID-19 who would have died in the year 2020 due to other, more common causes; and, less accidental deaths due to restrictions imposed to the public (such as regional lockdowns). Also, there was no method to precisely attribute the mid-year number of excess deaths, as COVID-19 deaths do not occur at a constant rate, but in “waves.”

Despite these limitations, these results provide quantitative insight on how COVID-19 has affected the United States in 2020. With the continued escalation of the coronavirus in 2021 until sufficient levels of vaccination have been achieved, this may better educate the public about the severity of the disease, and how the pandemic has affected the entire population (not just the elderly) due to both the direct and indirect effects of COVID-19. Additionally, this may inform decision makers considering the most appropriate course of action with limited healthcare resources. Lastly, as states have been affected by the disease differently, the online app can be used to observe how COVID-19 impacted each respective state in the year 2020 and can further shed light on the impact and the detrimental damage that the disease could incur on the health of the population this upcoming year in 2021 if the number of deaths due to COVID-19 continue in an upwards trajectory.

## Supporting information

S1 Appendix Net impact of COVID-19: Excess deaths and total years of life lost: Online appendix. (PDF)
