# Supplementary material for: Impact of COVID-19 on excess mortality, life expectancy, and years of life lost in the United States
Source: PLoS One. 2021 Sep 1;16(9):e0256835. doi: 10.1371/journal.pone.0256835 (PMC8409675; doi:10.1371/journal.pone.0256835)
Supplement: S1 Appendix — (PDF) [file pone.0256835.s001.pdf]

Direct vs Indirect Impact of COVID-19 in the United States

Contents

Direct vs Indirect Impact of COVID-19 in the United States 1
List of Figures 1
List of Tables 2
Introduction 3
Imputing Suppressed Data from the CDC 3
Underlying Cause of Death (1999 - 2019) 3
Provisional COVID-19 Death Counts by Sex, Age, and State 5
Excess Mortality 5
Establishing a Baseline Number of Deaths 6
Excess Mortality attributed to COVID-19 6
Excess Mortality due to Direct vs Indirect Effects of COVID-19 7
Life Expectancy 8
Reference Life Expectancies 9
Approximating Life Expectancy in the United States 9
Estimating the Number of Years Lost in Historical Life Expectancy Gains 12
Estimating the Number of Years Lost in Life Expectancy 12
Years of Life Lost (YLL) attributed to COVID-19 15
Direct vs. Indirect Effects of COVID-19 on YLL 16
References 17

List of Figures

Figure 1: Total excess mortality in 2020 for 50 states in the United States. 7
Figure 2: Excess mortality due to direct vs indirect effects of COVID-19. Errorbars indicate the lower and upper bounds of the plausible range of excess mortality. 8
Figure 3: Reference life expectancy at birth in the United States. 10
Figure 4: Life expectancy at birth in the United States during the 2020 COVID-19 pandemic 11
Figure 5: Number of years in life expectancy lost attributed to COVID-19 in the United States in 2020. 12
Figure 6: The number of years lost in life expectancy in the United States. 13
Figure 7: Number of years lost in life expectancy due to direct and indirect effects of COVID-19 on the female, male, and total population of the United States. Error bar is the uncertainty of the net effects of COVID-19 for each population. 14
Figure 8: Years of life lost due to COVID-19 in the United States. 15
Figure 9: Years of life lost due to COVID-19 in the total population of the United States 16

List of Tables

Table 1: Number of years lost in historical life expectancy gains. 18

Introduction

More than 300000 deaths in the United States have been attributed to COVID-19 in the year 2020, with continued escalation expected in 2021 until sufficient levels of vaccination have been achieved. Given the magnitude and scale of mortalities, it is important to provide objective and meaningful measures of the impact of COVID-19 across the population of the United States.

To quantify the net public health impact of COVID-19 in the United States, we calculated the impact on the following three metrics: excess mortality, life expectancy, and years of life lost (YLL). In this manuscript, not only are we interested in how the number of deaths attributed to COVID-19 have affected these metrics on the health of the population (i.e. the direct effect), but we are also interested in whether the indirect effects of COVID-19, such as reduced access to healthcare due to diversion of healthcare resources, has also contributed to the decrease in the country's population health. In this manuscript, we attempt to quantify the direct and indirect effects of COVID-19 in the United States.

Now that the Centers for Diseases Control and Prevention (CDC) (1) has released the provisional number of deaths that occurred up to the end of 2020, we can approximate life expectancies for each age and sex strata of United States population, and for each of its 50 states. (The reason why we say "approximate" rather than "calculate" despite knowing the number of deaths is because we still need to estimate the mid-year population, since the official numbers have not been released as of writing this manuscript.) However, since we are interested in quantifying how COVID-19 has impacted life expectancy, we also need reference life expectancy values to which we can compare our approximate life expectancies. These reference life expectancy values are approximations of what the life expectancies would have been in the year 2020 if COVID-19 had not occurred. We also use these reference values to determine the number of years of life lost in the United States (and at the state level) in the year 2020.

In the following, we provide our methodology in detail for approximating these metrics. This manuscript is organized as follows. We first provide the methodology on how we imputed suppressed data values from data sets provided by the CDC. Then, we provide the methodology and results for excess mortality, impact on life expectancy, and total years of life lost across each strata of the population. All the code and additional tables used to produce results presented in this manuscript can be found in our GitHub Repository: https://github.com/MEDICI-UWO/Impact-of-COVID-19-on-the-US.

Imputing Suppressed Data from the CDC

In accordance with National Center for Health Statistics (NCHS), cells with counts that are between 1-9 are suppressed for all CDC data sets. In our analysis, we used two data sets from the CDC: "Underlying Cause of Deaths (1999 - 2019)" and "Provisional COVID-19 Deaths Counts by Sex, Age, and State".(1,2) In the following, we provide the methodology and our assumptions for imputing missing data for both data sets.

Underlying Cause of Death (1999 - 2019)

The main purpose of using this data set was to extrapolate the number of deaths that would have potentially occurred in the year 2020 if COVID-19 had not entered the population. We used these extrapolated values for various calculations, including excess mortality (COVID- and non-COVID related), the reference life expectancies, and to determine the population in the year 2020 for the United States. Since the main purpose of using this data set was to extrapolate the number of deaths, we did not

require all values to be filled-in. Therefore, we only imputed enough data cells to extrapolate the potential number of deaths that would have occurred in the year 2020 for each desired group, which allowed us to minimize the need for missing data assumptions.

In this manuscript, since we aim to inform the impact of COVID-19 in the United States not only at the country level, but also the state level stratified age and sex, we required the data set to be grouped by state, sex, age groups and year. For our analysis, we decided to use ten-year age groups (< 1 year, 1-4 years, 5-14 years, 15-24 years, 25-34 years, 35-44 years, 45-54 years, 55-64 years, 65-74 years, 75-84 years, and 85+ years) rather than five-year age groups or single-year ages for two reasons: 1) by grouping more people into one age group, we hoped that there would be less suppressed data cells, and 2) the "Provisional COVID-19 Death Counts by Sex, Age, and State" data set provides data for the ten-year age groups, so by using the same age groups, we are able to calculate our desired metrics by age groups as well.

Since this data set was exported from CDC Wonder, we were able to group the results in various ways to help us logically impute the suppressed data with few assumptions. We first grouped the data set by state, age group, and year; this provided the total (both male and female) number of deaths for this grouping, which allowed us to calculate any suppressed data cells in which their corresponding data cells, but of the opposite sex, was known. Additionally, if the total number of deaths was 18, we concluded that the value for both sexes would have to be 9 if both values were suppressed, as both values would have to be less than 10. Then, we grouped the data set by state and year; this enabled us to calculate the total number of deaths that occurred in an age group if it is the only age group in the state and year that was suppressed. However, since every state has unclassified data points in which the age groups are not stated (which, unfortunately, are all suppressed), we made the assumption that if the difference between the sum of all the deaths in our incomplete data set and the value given in the regrouped data set was less than ten, we would accept this value as the total number of deaths that occurred for the age group (for which the data cell was suppressed) in the given state and year. Even though this assumption would result in a slight overestimation for these particular values, given that the values of unclassified data points are very small, the error for the imputed values would also be very small. Thirdly, we grouped the data set by state, sex, and year; this was to identify both the suppressed values of male and female deaths in a given state and year if the total number of deaths of the corresponding groups is known. We calculated the difference in the total number of male and female deaths for each state and year between the original data set and the new data set (grouped by state, sex, and year) separately, and compared the sum of the resulting values to the total number of deaths for the corresponding state and year. If the values matched, we would assume that the differences calculated for the male and female population would be the number of deaths for their respective sex. However, using this grouping does not impute all suppressed data cells in which the total number of deaths for the corresponding state and year are known. Further, this also does not provide us enough data points to extrapolate the data according to our desired grouping. We, therefore, impute these data cells by using the average proportion of male and female deaths in the United States for their corresponding age groups. Lastly, since as mentioned earlier, the data values for cells that correspond to an unspecified age group are very small, to reduce the number of variables, we removed these values from the data set.

Provisional COVID-19 Death Counts by Sex, Age, and State

Since this data set provides not only the number of deaths grouped by ten-year age groups, sex, and state, but also gives additional data points such as the total number of deaths by sex and state and the number of deaths by age groups and sex in the entirety of the United States, similar to what we did to the previous data set "Underlying Cause of Deaths (1999 - 2019)", we were able to group the data set in ways that allowed us to impute suppressed data cells through logic. However, unlike the previous data set, we require all suppressed data values to be imputed as every data value is required for our analysis. This resulted in the addition of a few assumptions, outlined below. Note that this data set provides both the number of deaths attributed to COVID-19 and the total number of deaths, and the methodology outlined here applies to both.

We first imputed the suppressed data cells for states and sex in which we were only missing data from one age group. This was accomplished by taking the sum of the number of deaths (both COVID-19 and total) for each group (by state and sex) and subtracting the results from the total number of deaths, which is also given in the same data set. Additionally, using the same grouping, we were able to impute suppressed data values in which the difference between the sum and the given total values were equal to the number of missing data values; this meant that each of the data values were equal to one. Then, we imputed the missing data cells for age groups and sex in which we were only missing data from one state; we took the sum of the number of deaths within each group and compared the results to the given values corresponding age group and sex in the United States. Unfortunately, this does not impute all of the suppressed data cells, and therefore, we need to introduce some assumptions to fill in the rest of the data set.

To impute the rest of the suppressed data values, for each sex within each state, we used the number of deaths that occurred for the corresponding sex in the United States, and took the ratio between the age groups with missing data values and multiplied the ratios with the number of deaths that have not been accounted for (i.e. the difference between the sum of the number of deaths and the recorded total number), which we then round to the nearest integer. Since the unaccounted deaths were small (since for a value to be suppressed, it needs to be less than 10), multiplying the ratio would occasionally result in a value of 0, which we know would not be the case. For this scenario, we added one to these data cells and subtracted one from the largest value within the missing group (to maintain the same number of deaths as the reported total). Conversely, we also calculated values that were greater than 9. As there is no way for us to determine where the excess deaths should be allocated (especially for states with more than 2 missing data values), we decided to leave the imputed value as is, despite knowing that this is an over approximation. Lastly, due to rounding error, the sum of the imputed values does not always equal to the unaccounted deaths; for this, we also decided to leave the imputed values as what was calculated, as the results would not materially change with further manipulation.

Since the original data set also contains data values corresponding to an unknown sex for each state, we excluded these values for the male and female population but included these values for the total population.

Excess Mortality

Excess mortality refers to the number of deaths from all causes due to a crisis (such as a pandemic) that is beyond what we would have expected to see under "normal" conditions.(3) There are several ways to calculate excess mortality: one method is to compare the number of deaths that occurred in the year of

interest (in this case, 2020) to the mean number of deaths reported for the corresponding week over the previous five years (which numerous researchers(4–6) have done to estimate excess mortality); another method is to use the Farrington surveillance algorithms to determine the number of excess deaths (which is what the CDC(7) uses). We decided to take a more comprehensive approach to calculate excess mortality: we compared the reported deaths to the extrapolated result of the number of deaths (grouped by state, sex, and age group) based on the trend that occurred in the previous 21 years, as follows.

Establishing a Baseline Number of Deaths

In order to estimate excess mortality from all causes, we first needed to establish a baseline for the number of 2020 deaths that would have been expected under "normal" conditions. We estimated the number of deaths that would have potentially occurred in the year 2020 (without COVID-19) by linearly regressing the reported annual number of deaths from 1999-2019, retrieved from CDC Wonder(2) and imputed (see Imputing Suppressed Data from the CDC section), for every combination of age group, sex, and state in the United States. To account for the uncertainty of our estimate, we used the 95% prediction interval to establish the lower and upper bound of our estimate. As it is impossible to have negative deaths, we changed all negative results to 0.01. The reason why we replaced negative values with 0.01 instead of 0 is because later, we used these number of deaths to calculate the life expectancy. If any values representing deaths for an age cohort is equal to zero, the life expectancy cannot be calculated due to the formula which requires dividing by the death rate (which would be zero if the number of deaths is zero). The baseline number of deaths for each respective group that we used in our analysis is given in our GitHub repository.

Excess Mortality attributed to COVID-19

After estimating the baseline number of deaths, we calculated the excess all-cause mortality in 2020 by subtracting the baseline number of deaths that we estimated by extrapolation from the total deaths reported by the CDC corresponding to each respective group (by sex, state, and age group). We also calculated the lower and upper bound for the excess all-cause mortality, where the difference between the reported number of deaths and the lower bound of our baseline estimate resulted in the upper bound of the excess all-cause deaths, and conversely, the difference between the reported number of deaths and the upper bound of our baseline estimate gave the lower bound for excess all-cause deaths. Figure 1 shows the total number of excess mortality attributed to COVID-19 in each state in the United States. To calculate the total number of excess mortality, we took the sum of the excess deaths for all the age groups according to sex and state. In the figure, we observe that New York was the state with the most excess mortality. On the other hand, North Carolina has the least number of excess mortality, which is also negative. Negative excess mortality indicates that less deaths occurred than what we expected despite the pandemic, suggesting that there are other factors that decreased the number of

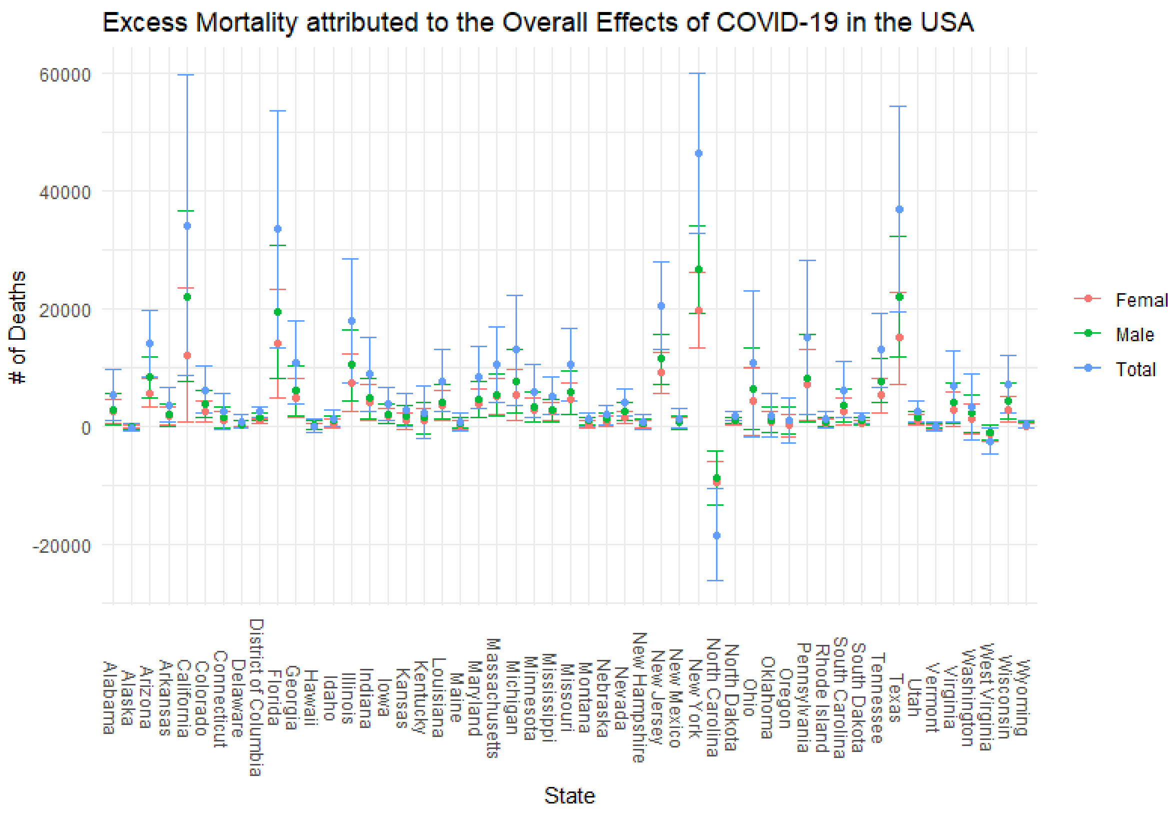

Figure 1: Total excess mortality in 2020 for 50 states in the United States.

deaths due to more common causes. We explore this idea in our next section when comparing direct and indirect effects of COVID-19.

Excess Mortality due to Direct vs Indirect Effects of COVID-19

In addition to estimating the excess mortality attributed to COVID-19, we are interested in how the direct and indirect effects of this disease attributed to these deaths separately. In this manuscript, we consider all COVID-19 deaths reported by the CDC as excess mortality due to the direct effects of COVID-19. On the other hand, the number of excess mortality due to indirect effects is calculated by taking the difference between the reported non-COVID-19 deaths (total deaths minus COVID-19 deaths) and the baseline number of deaths. Due to our assumption in which we have defined all COVID-19 deaths as excess mortality due to the direct effects of COVID-19, this occasionally results in the number of excess mortality due to the indirect effects of the disease to be negative. A few possible reasons why we see negative indirect effects include the possibility that some COVID-19 deaths were not correctly diagnosed and reported, a number of people who died from COVID-19 who would have died in the year 2020 due to other, more common causes, and less accidental deaths due to restrictions imposed to the public (such as regional lockdowns). Figure 2 compares the number of excess mortality due to the direct and indirect effects of COVID-19 for the total population of the United States by age groups. We observe that those between the ages of 5 and 44 are heavily affected due to indirect effects of COVID-19 in comparison to the direct effects. On the other hand, those who are above the age of 44 are more affected by COVID-19 itself rather than other causes of death. Altogether, the United States had a total of 375235 (95% prediction interval: (132380, 618088)) excess mortality due to the effects of the

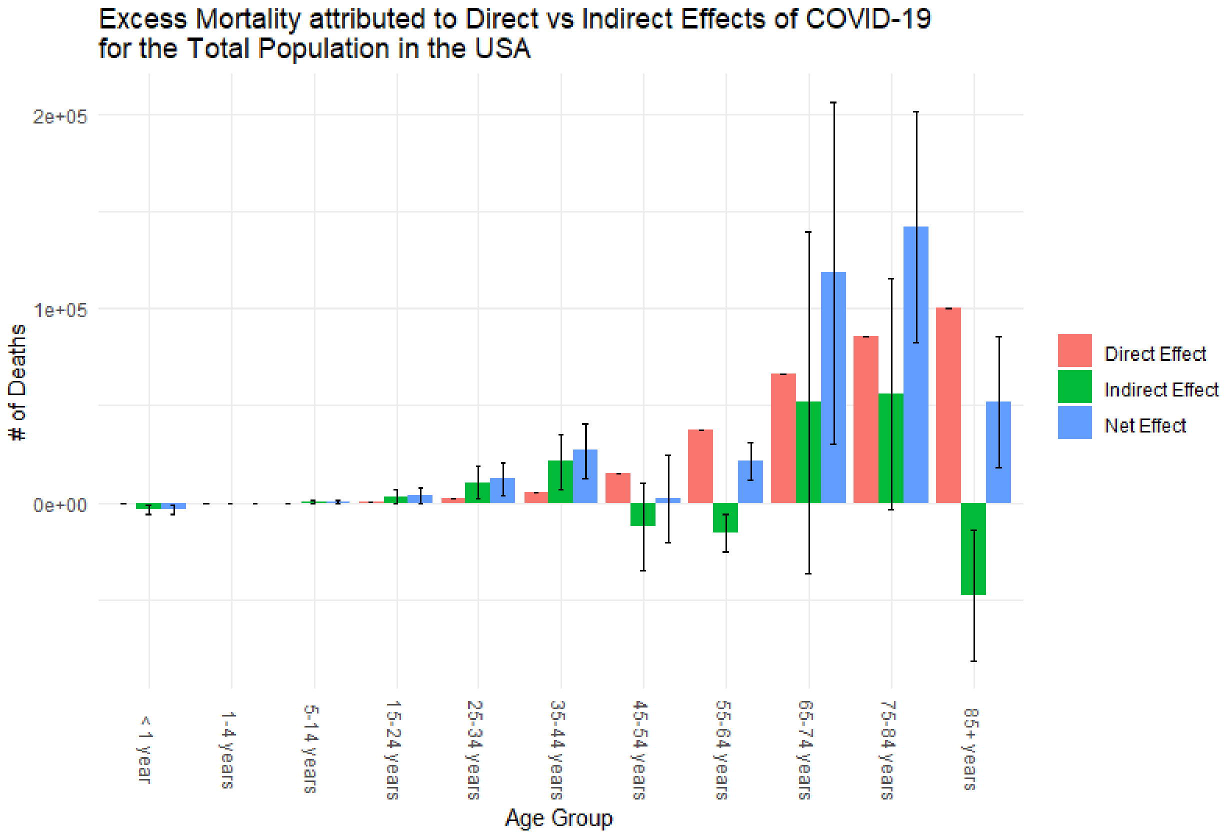

Figure 2: Excess mortality due to direct vs indirect effects of COVID-19. Error bars indicate the lower and upper bounds of the plausible range of excess mortality.

pandemic, 313171 of which are due to the direct effects and 62064 (95% prediction interval: (-180791, 304917)) of which are due to the indirect effects of COVID-19.

Life Expectancy

A life table is a data-driven tool that summarizes the probability of a person (of a certain age) dying before their next birthday (this can also be done with age groups rather than single year age). These probabilities allow us to calculate the life expectancy at birth via a series of calculations represented in columns in the life table.

In this manuscript, we calculated the decrease in life expectancy at birth due to the increase number of deaths due to the COVID-19 pandemic in the United States in the year 2020. As some states were hit with the virus more severely than others, not only did we calculate the decrease in life expectancy for just the United States as a country, but we also calculated the decrease in life expectancy for each state, and for the male and female population separately. In addition to calculating the overall decrease in life expectancy, we also explored how much of this decrease was attributed to the direct and indirect impact of COVID-19, separately.

For all life expectancy calculations in this manuscript, we used abridged life tables by ten-year age groups (< 1 year, 1-4 years, 5-14 years, 15-24 years, 25-34 years, 35-44 years, 45-54 years, 55-64 years, 65-74 years, 75-84 years, and 85+ years). For details on calculating life expectancy using an abridged life table, see the Measure Evaluation website.(8)

Reference Life Expectancies

In order to find the decrease in life expectancy that occurred in the year 2020 attributed to COVID-19, we first needed to establish a reference life expectancy that we can compare our results to. This reference life expectancy is an estimate of what the life expectancy of the United States and its respective 50 states would have been under "normal" circumstance (i.e. if the pandemic had not occurred). To calculate our reference life expectancy, we extrapolated the number of deaths and the mid-year population to estimate what the values would have been under "normal" conditions and using these values, we calculated the life expectancy with an abridged life table. Since we have already estimated the number of deaths that would have occurred when determining the number of excess mortality, we direct you to the Excess Mortality section for the methodology since we used the same values in this section.

Estimating the Mid-Year Population

To estimate the mid-year population for the year 2020, we regressed the (estimated) mid-year population data set from 1990 to 2019 (retrieved from CDC Wonder(9)) using a cubic polynomial. We regressed the data set for every combination of age group, sex, and state. Similar to what we did when we extrapolated the number of deaths, we also calculated the 95% prediction interval to provide a range of plausible values for each grouping. For the mid-year population that we used for the United States and each individual states, see our GitHub repository.

Calculating the Reference Life Expectancies

Using the extrapolated values of the mid-year population and number of deaths, we used an abridged life table to calculate the reference life expectancy for each state and by age group for the total, male, and female population, separately. Since we also provided the lower and upper bound when estimating the number of deaths and the mid-year population, not only did we calculate the life expectancy corresponding to best fit, we also used the lower and upper bounds to also give a plausible range in which the reference life expectancy could be. For the fitted life expectancy value, we used the fitted value for both the mid-year population and the number of deaths. To calculate the lower bound of life expectancy, we used the upper bound of the estimated annual number of deaths and the lower bound of the mid-year population (this combination gives the greatest possible death rate for each age group, and therefore, gives the lowest life expectancy, thus the lower bound). Conversely, to calculate the upper bound of life expectancy, we used the lower bound of the number of deaths and the upper bound of the mid-year population (this combination gives the lowest possible death rate for each age group, and therefore, gives the highest life expectancy, thus the upper bound). Figure 3 shows the reference life expectancy at birth (with the plausible range) for the total, male, and female population for each state in the United States.

Approximating Life Expectancy in the United States

Similar to how we approached calculating the reference life expectancy, we also used an abridged life table to approximate what the life expectancy was in the United States during the pandemic. However, we use different values for both the number of deaths and the mid-year population in our calculations.

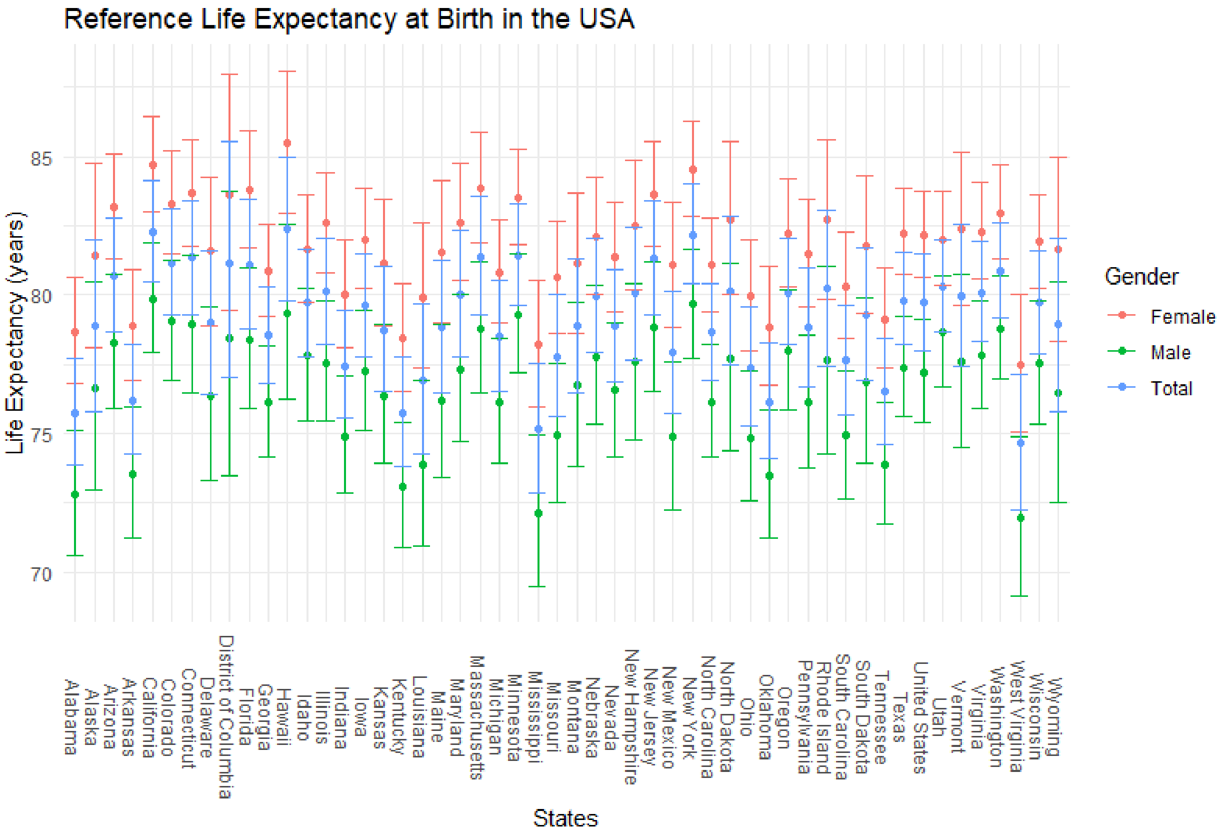

Figure 3: Reference life expectancy at birth in the United States.

Instead of approximating the number of deaths, we used the total deaths reported by the CDC. We also modify our mid-year population estimates to account for the excess mortality that occurred due to COVID-19.

Adjusting the Mid-Year Population

Since our estimated mid-year population that we used in our reference life expectancy calculations do not take the excess mortality due to the pandemic into account, we modified these values when approximating the life expectancy in the United States during the COVID-19 pandemic in 2020. This is particularly important as the decrease in mid-year population would result in the increase in the death rate, hence a shorter life expectancy. Since we are using the mid-year population, we cannot simply take the difference between the estimated mid-year population and the excess mortality that occurred in the year 2020, as this would underestimate the population (especially for groups with a large number of excess deaths). Instead, we used the excess mortality that we calculated previously in the Excess Mortality section and divided the values in half. Although we know that deaths due to COVID-19 occur in waves and not at a constant rate, this is a limitation that we have to accept as there is no way of precisely attributing the number of excess deaths we need to account for in the middle of the year. We subtracted these values from the estimated mid-year population from their respective group. Additionally, since we encountered negative excess mortality for some groups, for the mid-year population adjustment, we did not include any negative excess mortality in the calculation. Lastly, considering that we provided the fitted values, and the lower and upper bounds for both the mid-year population and the number of excess deaths, we can also provide a plausible range in which the

adjusted population could be. Calculating the fitted value is straightforward: we simply use the fitted value for both the mid-year population and the number of excess deaths. On the other hand, for the lower and upper bounds, we want to use a combination of values which would result in the least and greatest possible value, respectively. Therefore, for the lower bound of the adjusted mid-year population, we take the lower bound of the extrapolated population and subtract it by the upper bound of the number of excess mortality. In contrast, for the upper bound, we take the upper bound of the extrapolated population and subtract it by the lower bound of the number of excess mortality.

Approximating the Life Expectancy in the United States

Using the total deaths reported by the CDC and the adjusted mid-year population, we used the abridged life table to approximate the life expectancy during the year 2020 across the United States, and for the individual 50 states, respectively. In addition to calculating the life expectancy for the total population, we also calculated the life expectancy for the male and female population separately. Lastly, similar to our approach to calculating the reference life expectancy, we also provided the lower and upper bound of the plausible range in which the life expectancies could potentially be. However, since we do not have a range for the number of deaths to consider as the exact numbers are given, the lower and upper bounds of the life expectancy are only dependent on the mid-year population. To calculate the lower bound of the life expectancy, we used the lower bound of the mid-year population, and to calculate the upper bound of the life expectancy, we used the upper bound of the mid-year population. Figure 4 shows what we approximated the life expectancy to be for the total, male, and female population of the United States (at both the country and state level). Additionally, we want to note that we could not

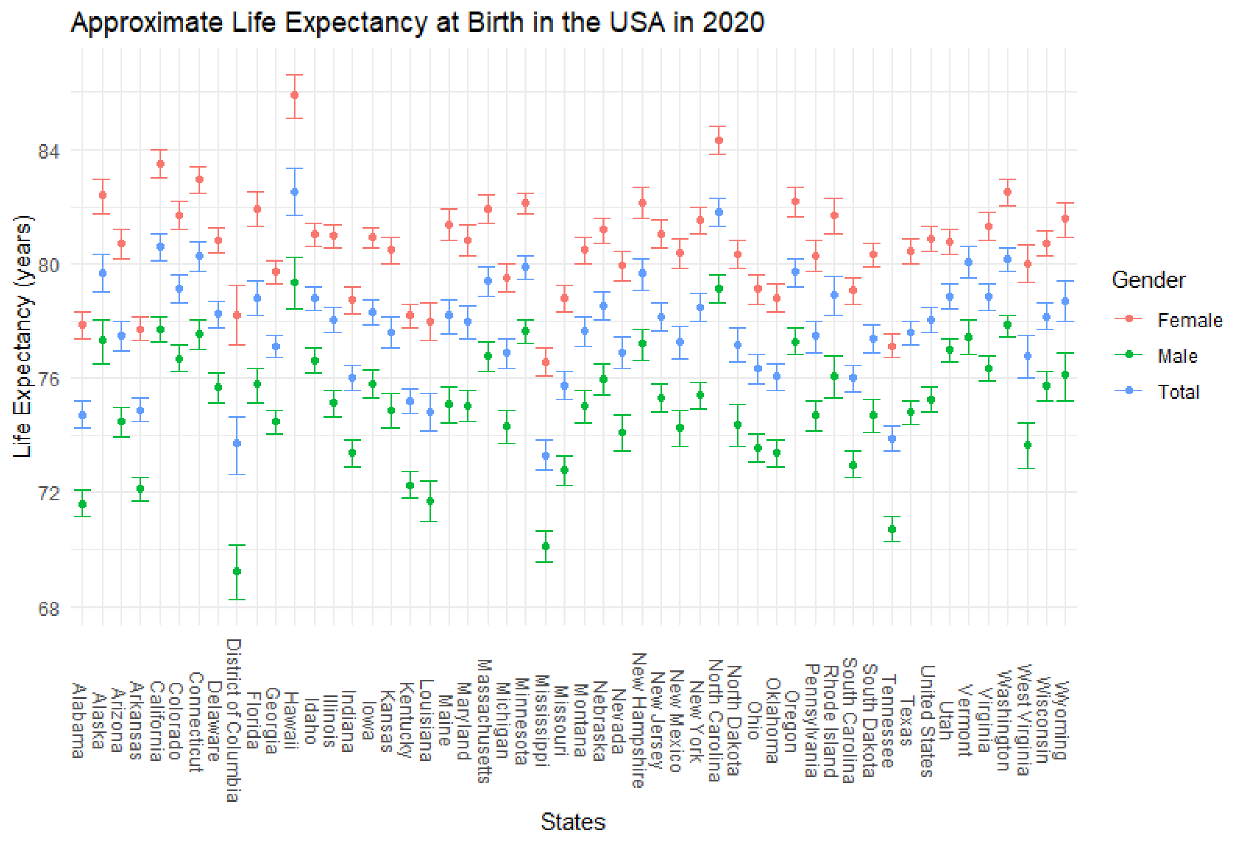

Figure 4: Life expectancy at birth in the United States during the 2020 COVID-19 pandemic

calculate the life expectancy for the female population of Vermont since the number of deaths for one of the age groups was zero.

Estimating the Number of Years Lost in Historical Life Expectancy Gains

Using the approximated life expectancies (at birth), we also estimated the number of years lost in historical life expectancy gains. Using the mid-year populations from the “Bridged-Race Population Estimates” data set(9) and the imputed version of the number of deaths from the “Underlying Cause of Death” data set(2), we calculated the life expectancy in the United States and its 50 states from 1999 to 2019 using an abridged life table. We then compared the life expectancy for each respective region and sex from 2020 to the corresponding list of historical life expectancy values. This allowed us to determine the previous time the life expectancy was at a similar value to the one in 2020, by finding the most recent year in which the life expectancy was less than the one that we had calculated for 2020. If there are no historical life expectancy values that is less than what was approximated for the year 2020, then we conclude that the year would have to be earlier than 1999 (which is the earliest year that we were able to calculate the life expectancy for). Subtracting the year that we had matched from 2020 gave us the number of years lost in historical life expectancy gains. The results are given in Table 1.

Estimating the Number of Years Lost in Life Expectancy

In addition to estimating the number of years lost in historical life expectancy gains, we are able to calculate the number of years lost in life expectancy now that we have approximated the actual life expectancies and their respective reference values. We calculate the decrease in life expectancy in the

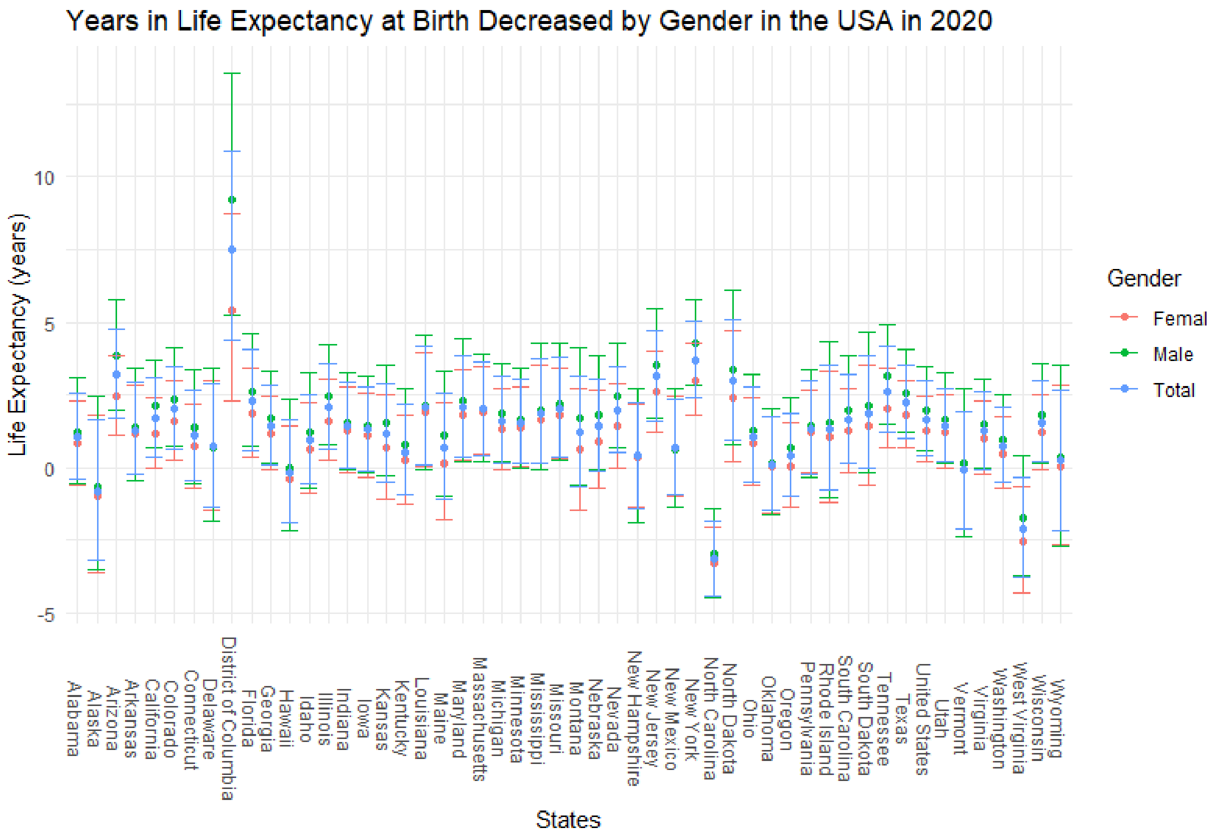

Figure 5: Number of years in life expectancy lost attributed to COVID-19 in the United States in 2020.

year 2020 by taking the difference between the two values with respect to the fitted values, and the lower and upper bounds. The results indicate the impact that the direct and indirect effects of COVID-19, together, had on life expectancy. Figure 5 presents the number of years in life expectancy lost due to COVID-19 in the United States.

However, we are also interested in identifying what portion of this life expectancy decrease is attributed to the direct and indirect effects separately. In the following, we explain our methodology to calculate this.

Life Expectancy Decrease Attributed to Direct vs Indirect Effects of COVID-19

As we have stated in the Excess Mortality section, we assumed that all reported COVID-19 deaths are excess mortality attributable to the direct effects of the virus. Therefore, to estimate the decrease in life expectancy in the United States due to the indirect effects of COVID-19, we used the abridged life table for the third time to approximate the life expectancy with no COVID-19 deaths. For this calculation, we used the number of non-COVID-19 deaths reported by CDC (total deaths minus COVID-19 deaths) and the estimated mid-year population (which we used in our reference life expectancy calculations, see the Reference Life Expectancies section for details). Again, we also provided a range of plausible values for the life expectancies; similar to our approach in approximating the lower and upper bounds of the "actual" life expectancies in the United States, the lower and upper bounds are only dependent on the mid-year population, in which we use the lower and upper bounds, respectively. The difference between the reference life expectancy and the corresponding non-COVID-19 life expectancy is what we consider as the number of years lost in life expectancy due to the indirect effects of COVID-19. As

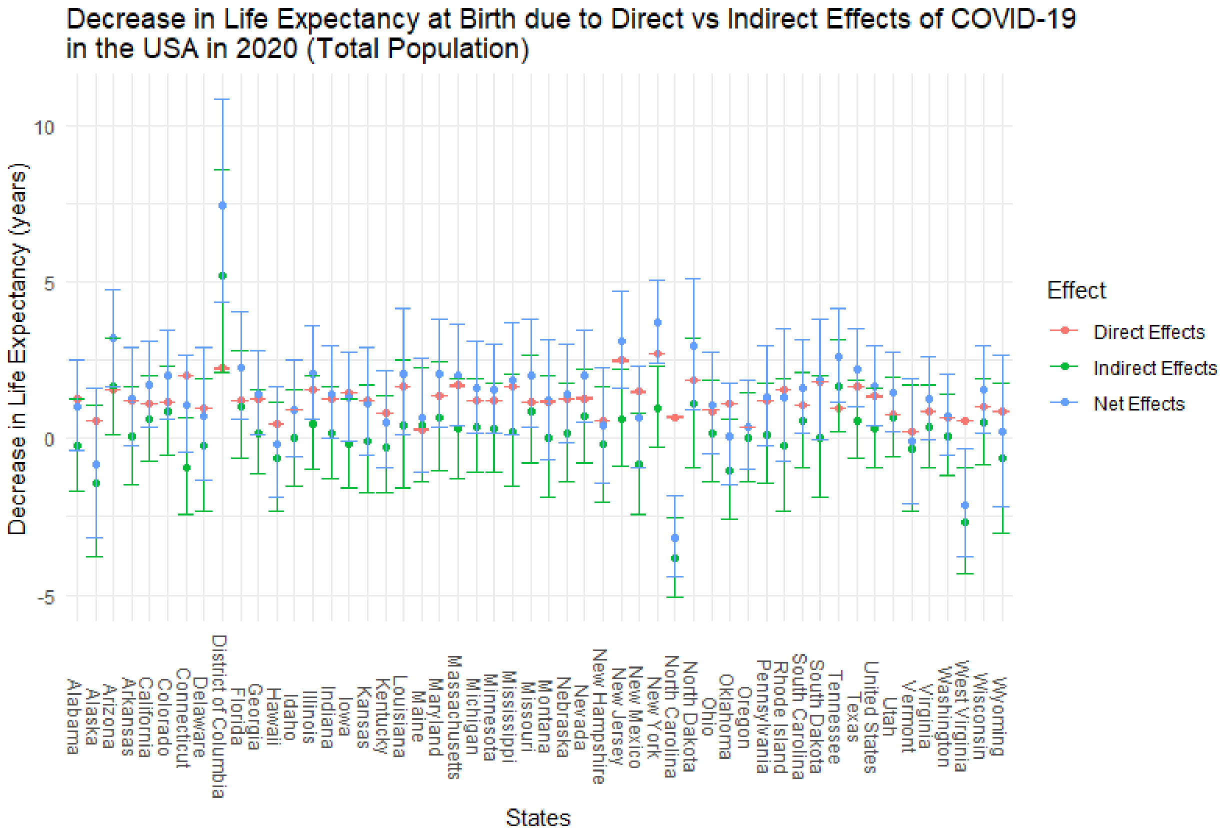

Figure 6: The number of years lost in life expectancy in the United States.

mentioned above, due to our assumption in which we have defined all reported COVID-19 deaths as excess mortality attributable to the direct effects of COVID-19, this occasionally results in the number of excess deaths due to the indirect effects of the disease to be negative, resulting in a negative decrease (i.e. an increase) in our life expectancy calculations. We provided a few possible explanations as to why this may be the case in the Excess Mortality section.

On the other hand, to calculate the decrease in life expectancy attributed to the direct effects of COVID-19, we took the difference between our approximation of the life expectancy, which we calculated in the Approximating Life Expectancy in the United States section of this manuscript and the non-COVID-19 life expectancy for each corresponding group. Since the "actual" life expectancies are calculated based on deaths due to both the direct and indirect effects of COVID-19, and the non-COVID-19 life expectancies are calculated based on only the indirect effects of the disease, the difference between the two calculated life expectancies should result in the number of years lost in life expectancy attributable to the direct effects of COVID-19.

Figure 6 compares the number of years lost in life expectancy due to direct and indirect effects of COVID-19 for the total population in the United States and its 50 states. We also compared the direct and indirect effects of COVID-19 on the male and female population in Figure 7. This figure clearly shows that the direct effects of COVID-19 is the predominant factor in the decrease in life expectancy in the United States.

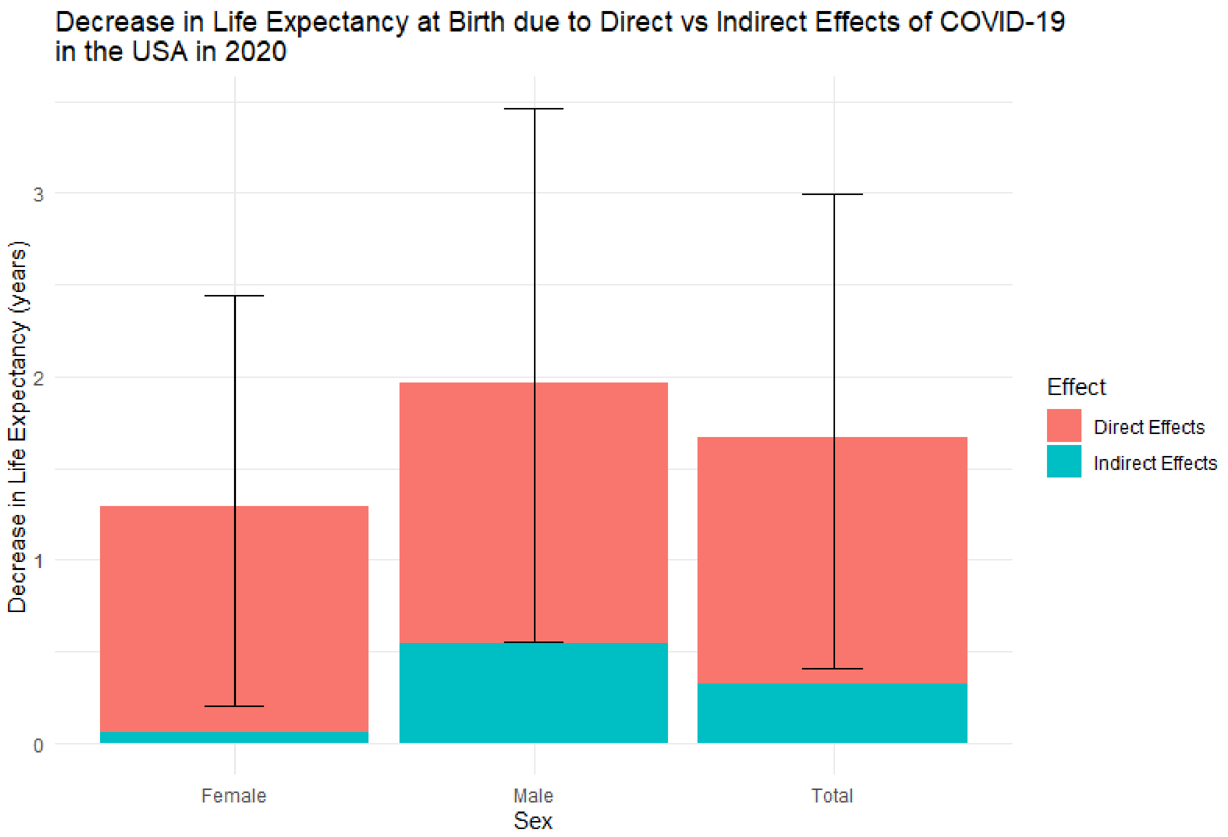

Figure 7: Number of years lost in life expectancy due to direct and indirect effects of COVID-19 on the female, male, and total population of the United States. Error bars are the uncertainty of the net effects of COVID-19 for each population.

Years of Life Lost (YLL) attributed to COVID-19

In addition to the excess mortality and the number of years lost in life expectancy, we are also interested the years of life lost (YLL) in the United States attributed to COVID-19. YLL estimate the years of potential life lost due to premature death, taking the age at which deaths occur into account. This means that greater weight is given to deaths at a younger age and lower weight to deaths that occur at an older age.(10) Years of life lost are calculated by multiplying the number of deaths with a standard life expectancy at the age at which death occurs. To estimate what the life expectancy would have been in the United States under "normal" conditions, we used our reference life expectancy values from the previous section. Additionally, rather than using the number of deaths attributed to COVID-19 to calculate the total years of life lost for each state, we used the excess mortality due to both the direct and indirect effects of COVID-19, as this provides a better depiction of the net impact of COVID-19 for the population, including all deaths above the expected number of deaths (both those reported as COVID-related and those not COVID-related) under "normal" circumstances. Figure 8 shows the YLL attributed to COVID-19 for all 50 states in the United States. This figure, however, does not necessarily provide any new information than what we have seen in Figure 1, as the distribution of deaths across age groups are quite similar for most states. Therefore, it is more interesting for us to look at the YLL for each age cohort, which we will encounter in the following section when examining what portion of the YLL are attributed to direct and indirect effects of the disease.

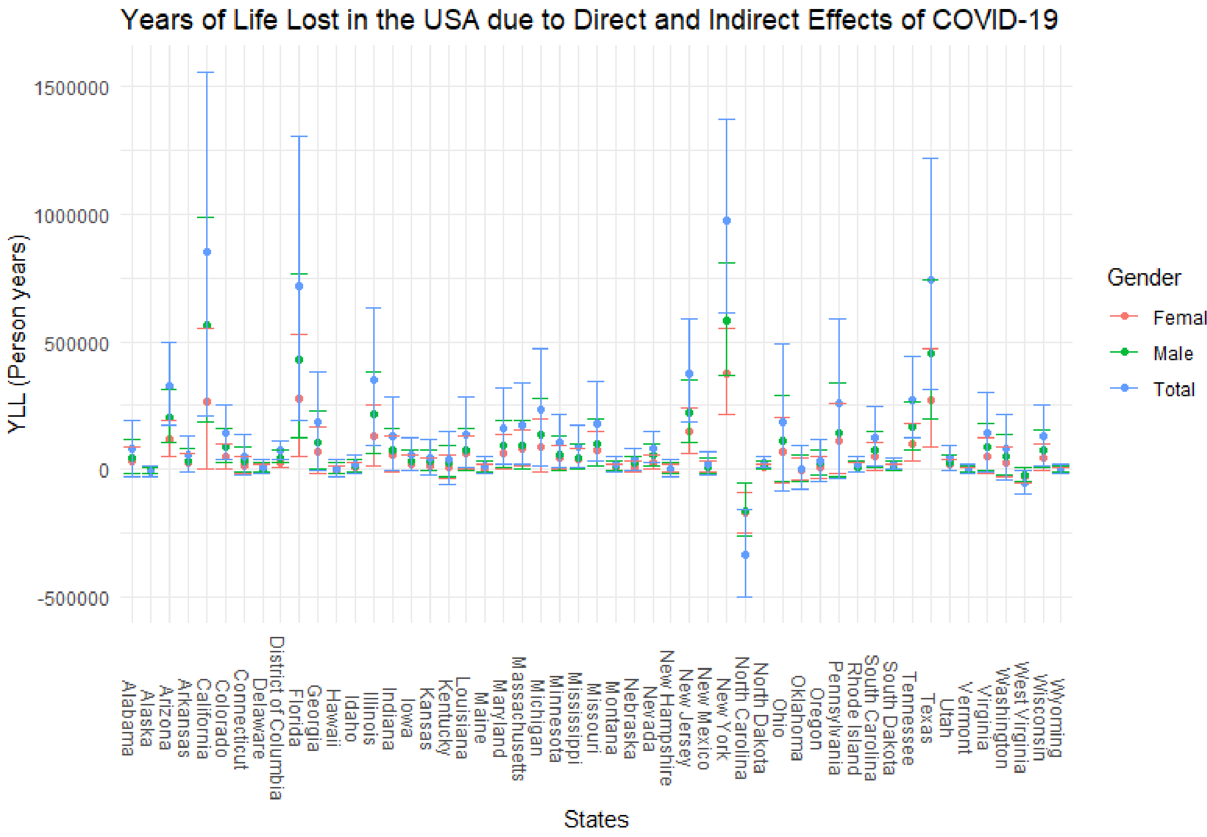

Figure 8: Years of life lost due to COVID-19 in the United States.

Direct vs. Indirect Effects of COVID-19 on YLL

To determine the YLL that are attributed to the direct and indirect effects of COVID-19 separately, we used the number of excess deaths attributable to direct and indirect effects of COVID-19 that we established in the Excess Mortality section of this manuscript. To reiterate, we considered all deaths reported as related to COVID-19 as excess deaths due to the direct effects of the virus, and any additional deaths due to other causes as excess mortality due to indirect effects. Using the excess deaths that we calculated previously, we multiplied these values with the number of years remaining (taken from the reference life expectancies) corresponding to their respective age group.

Figure 9 compares the YLL attributed to direct and indirect effects of COVID-19 in the total population of the United States. We observe from this figure that despite less weight given to the older age groups, the YLL for those who are 65 and above contribute to the majority of the years of life lost in the total population of the United States. Additionally, the YLL emphasize the number of excess deaths due to the indirect effects for those between the ages of 5 and 44. Despite COVID-19 heavily affecting the older population, this result suggests that the younger population is also being heavily affected during the pandemic, but indirectly. Lastly, the YLL attributed to COVID-19 in the United States is approximately 7,362,555 person-years (95% prediction interval: (1,596,202, 13,669,696)): 5,340,469 (5,068,888, 5,627,895) of those YLL is directly attributed to COVID-19, whereas 2,022,086 (-3472687, 8041802) person-years are indirectly attributed (collateral effects leading excess deaths during the pandemic).

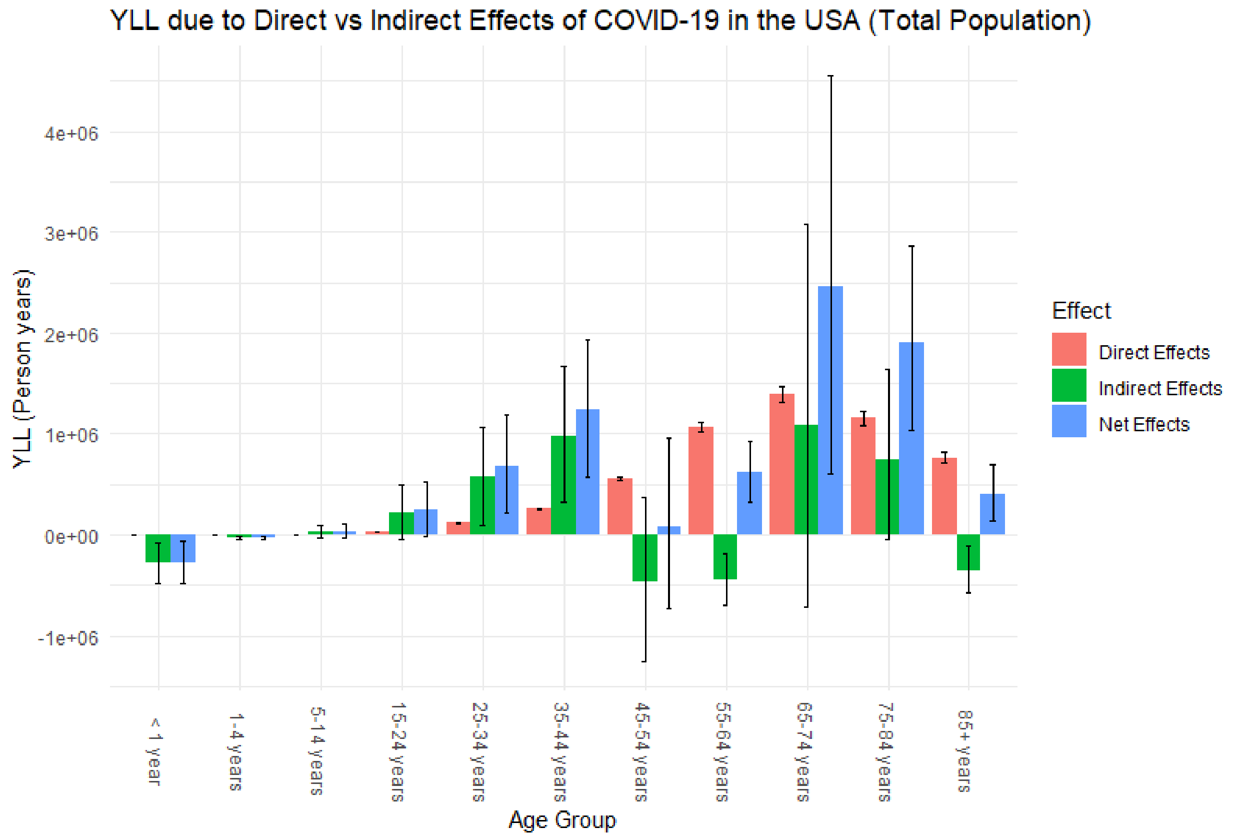

Figure 9: Years of life lost due to COVID-19 in the total population of the United States

References

1. National Center for Health Statistics. Provisional COVID-19 Death Counts by Sex, Age, and State | Data | Centers for Disease Control and Prevention [Internet]. [cited 2021 Jan 6]. Available from: https://data.cdc.gov/NCHS/Provisional-COVID-19-Death-Counts-by-Sex-Age-and-S/9bhg-hcku

2. Centers for Disease Control and Prevention, National Center for Health Statistics. Underlying Cause of Death, 1999-2019 on CDC WONDER Online Database [Internet]. 2020 [cited 2021 Jan 3]. Available from: https://wonder.cdc.gov/ucd-icd10.html

3. Roser M, Ritchie H, Ortiz-Ospina E, Hasell J. Excess mortality during the Coronavirus pandemic (COVID-19) - Our World in Data [Internet]. 2020 [cited 2021 Jan 10]. Available from: https://ourworldindata.org/excess-mortality-covid

4. Oke J, Heneghan C. COVID-19 Mortality – ONS Update - CEBM [Internet]. [cited 2020 Jul 13]. Available from: https://www.cebm.net/covid-19/covid-19-mortality-ons-update/

5. Bernard S, Blood D, Burn-Murdoch J, Harlow M, Nevitt C, Smith A, et al. Coronavirus tracked: the latest figures as countries start to reopen | Free to read | Financial Times [Internet]. [cited 2020 Jul 13]. Available from: https://www.ft.com/content/a26fbf7e-48f8-11ea-aeb3-955839e06441

6. Estimates of the population for the UK, England and Wales, Scotland and Northern Ireland - Office for National Statistics [Internet]. [cited 2020 Jun 25]. Available from: https://www.ons.gov.uk/peoplepopulationandcommunity/populationandmigration/populationestimates/datasets/populationestimatesforukenglandandwalesscotlandandnorthernireland

7. Excess Deaths Associated with COVID-19 [Internet]. [cited 2020 Aug 31]. Available from: https://www.cdc.gov/nchs/nvss/vsrr/covid19/excess_deaths.htm

8. Lesson 3: Life Tables — MEASURE Evaluation [Internet]. [cited 2020 Jun 24]. Available from: https://www.measureevaluation.org/resources/training/online-courses-and-resources/non-certificate-courses-and-mini-tutorials/multiple-decrement-life-tables/lesson-3

9. United States Department of Health and Human Services (US DHHS), Centers for Disease Control and Prevention NC for HS. Bridged-Race Population Estimates, United States July 1st resident population by state, county, age, sex, bridged-race, and Hispanic origin. [Internet]. [cited 2020 Jul 27]. Available from: http://wonder.cdc.gov/bridged-race-v2019.html

10. World Health Organization. Years of life lost (percentage of total) [Internet]. [cited 2021 Jan 13]. Available from: http://whqlibdoc.who.int/bulletin/2005/Vol83

Table 1: Number of years lost in historical life expectancy gains.

Region	Total			Male			Female		
	fit	lwr	upr	fit	lwr	upr	fit	lwr	upr
United States	14	12	17	15	13	17	12	9	15
Alabama	14	4	21	14	12	20	12	3	15
Alaska	1	1	3	1	1	3	1	1	1
Arizona	18	15	>21	21	17	>21	15	14	>21
Arkansas	18	17	>21	17	15	>21	18	3	>21
California	12	9	13	13	12	14	7	3	11
Colorado	15	12	17	15	15	17	12	11	15
Connecticut	13	1	15	14	12	16	3	1	13
Delaware	2	1	15	2	1	15	8	2	15
District of Columbia	19	14	19	19	15	19	13	12	19
Florida	13	11	15	14	12	17	12	9	14
Georgia	11	5	13	12	10	13	10	3	12
Hawaii	1	1	11	1	1	11	1	1	5
Idaho	15	3	17	14	14	17	13	2	17
Illinois	14	12	17	15	14	17	12	11	14
Indiana	20	17	>21	20	17	>21	20	15	>21
Iowa	20	17	>21	20	17	>21	20	12	>21
Kansas	17	12	>21	18	15	>21	12	1	17
Kentucky	4	2	>21	17	3	>21	3	1	18
Louisiana	14	12	15	14	12	15	12	6	15
Maine	15	3	18	18	15	>21	3	2	5
Maryland	13	12	16	15	12	17	13	11	14
Massachusetts	15	13	17	16	13	17	14	12	16
Michigan	18	15	20	18	15	20	15	14	18
Minnesota	17	15	18	16	15	17	17	15	18
Mississippi	>21	15	>21	21	14	>21	18	15	>21
Missouri	21	17	>21	21	18	>21	17	15	>21
Montana	15	5	18	17	15	>21	12	3	17
Nebraska	17	12	18	17	15	18	13	5	17
Nevada	14	12	15	15	14	21	14	9	14
New Hampshire	2	1	15	2	1	4	2	1	13
New Jersey	17	15	19	19	17	19	15	14	17
New Mexico	17	1	>21	17	1	>21	17	4	>21
New York	18	17	19	19	18	21	15	14	17
North Carolina	1	1	1	1	1	1	1	1	1
North Dakota	>21	>21	>21	>21	>21	>21	>21	>21	>21
Ohio	18	3	>21	21	3	>21	17	2	21
Oklahoma	2	1	12	2	1	12	1	1	3

Oregon	3	1	12	5	1	12	1	1	3
Pennsylvania	15	3	18	15	3	18	15	3	17
Rhode Island	15	3	17	15	12	18	13	1	15
South Carolina	14	13	15	14	13	15	13	5	15
South Dakota	>21	17	>21	19	17	>21	>21	17	>21
Tennessee	>21	>21	>21	>21	>21	>21	>21	17	>21
Texas	15	12	17	16	15	17	12	10	15
Utah	16	14	>21	16	13	18	17	5	>21
Vermont	4	4	14	4	4	15	>21	>21	>21
Virginia	12	3	13	12	11	13	9	3	12
Washington	3	1	12	11	3	12	3	1	11
West Virginia	1	1	1	1	1	1	1	1	1
Wisconsin	18	17	21	18	15	21	18	15	>21
Wyoming	1	1	13	1	1	13	1	1	6
